# Real-world data analysis of survival outcomes of patients with primary mediastinal large B-cell lymphoma treated with immunochemotherapy: the role of consolidative radiation therapy

**DOI:** 10.1007/s44313-025-00076-4

**Published:** 2025-04-22

**Authors:** Yong-Pyo Lee, Junhun Cho, Young Hyeh Ko, Dongryul Oh, Seok Jin Kim, Won Seog Kim, Sang Eun Yoon

**Affiliations:** 1 Department of Internal Medicine, Division of Hematology-Oncology, Chungbuk National University Hospital, Cheongju, Korea; 2 Department of Pathology, Samsung Medical Center, Sungkyunkwan University School of Medicine, Seoul, Korea; 3 Department of Radiation Oncology, Samsung Medical Center, Sungkyunkwan University School of Medicine, Seoul, Korea; 4 Department of Medicine, Division of Hematology-Oncology, Samsung Medical Center, Sungkyunkwan University School of Medicine, Seoul, Korea; 5 Department of Health Sciences and Technology, Samsung Advanced Institute for Health Sciences and Technology, Sungkyunkwan University, Seoul, Korea

**Keywords:** Primary mediastinal large B-cell lymphoma, Front-line immunochemotherapy, Radiation therapy, Consolidative treatment

## Abstract

**Purpose:**

Primary mediastinal large B-cell lymphoma (PMBCL) is a rare subtype of diffuse large B-cell lymphoma. Radiation therapy (RT) has served as the primary treatment option for PMBCL; however, its role has been questioned with the advent of intensified immunochemotherapy. This study aimed to investigate the role of consolidative RT in the primary treatment of PMBCL.

**Methods:**

This single-center retrospective study analyzed the survival outcomes of 65 patients newly diagnosed with PMBCL. The patients were divided into three treatment groups: (1) EPOCH-R (etoposide, prednisone, vincristine, cyclophosphamide, doxorubicin, and rituximab), (2) R-CHOP (rituximab, cyclophosphamide, doxorubicin, vincristine, and prednisone), and (3) R-CHOP with consolidative RT.

**Results:**

The objective response and complete remission rates were 86.2% and 63.1%, respectively, with 3-year progression-free survival (PFS) and overall survival (OS) rates of 72% and 81%, respectively. All patients in the R-CHOP + RT group achieved an objective response with better PFS) than those who did not receive consolidative RT (*p* = 0.028), although there was no significant difference in OS (*p* = 0.102). Consolidative RT benefited patients with an initially bulky disease or insufficient end-of-treatment response. The predictive value of ^18^F-fluorodeoxyglucose positron-emission tomography-computed tomography (PET-CT) in assessing the treatment response in PMBCL was revalidated, showing that patients who achieved negative end-of-treatment PET-CT had significantly better survival outcomes than others.

**Conclusions:**

R-CHOP is a useful alternative regimen when intensified chemotherapy is not feasible. Consolidative RT should be considered in cases with an initially bulky disease and insufficient end-of-treatment response.

## Introduction

Primary mediastinal large B-cell lymphoma (PMBCL), a rare subtype of diffuse large B-cell lymphoma (DLBCL) involving transformed thymic B-cells, mainly affects the mediastinum. PMBCL has features that distinguish it from DLBCL. Clinical and molecular characteristics of PMBCL, such as activation of the Janus kinase-signal transducer and activator of transcription and nuclear factor-kB pathways, downregulation of major histocompatibility complex class I and II expression, as well as upregulation of programmed death ligands (PD-L) resemble those of Hodgkin lymphoma [1–3]. Owing to the low incidence of PMBCL, which accounts for < 5% of non-Hodgkin lymphomas, the clinical management strategies and treatment outcome predictions for PMBCL have been predominantly extrapolated from those used in DLBCL [4, 5]. Based on the accumulated data, front-line systemic treatment of PMBCL comprises CD20-targeted monoclonal antibody (such as rituximab) and anthracycline-containing regimens, such as CHOP (cyclophosphamide, doxorubicin, vincristine, and prednisone), EPOCH (etoposide, cyclophosphamide, doxorubicin, vincristine, and prednisone) and M/VACOP-B (etoposide or methotrexate, doxorubicin, cyclophosphamide, vincristine, prednisone, and bleomycin) [6, 7]. The 5-year progression-free survival (PFS) rates of these regimens are 78–81%, 87–93%, and 84%, respectively [3]. Despite these potent treatments, residual density makes it challenging to distinguish between fibrotic tissue and active lymphoma, and consolidative treatment is frequently required [4].

Considering the radiosensitive nature of PMBCL, implementation of upfront consolidative radiotherapy (RT) following front-line immunochemotherapy considerably decreases primary site relapse [4, 8, 9], making it a widely adopted practice. ^18^F-fluorodeoxyglucose positron-emission tomography-computed tomography (FDG PET-CT)-guided Lugano response assessment has emerged as an innovative methodology for identifying patients eligible for the omission of consolidative RT. Consequently, consolidative RT is recommended for patients with PBMCL who do not achieve complete remission (CR) as determined by PET-CT after front-line treatment [10, 11]. In addition, the introduction of various intensified immunochemotherapy regimens has undervalued the efficacy of consolidative RT [12, 13]. The absence of comparative analysis data within the confines of identical treatment protocols underscores the need for circumspection when evaluating the efficacy of consolidative RT.

We conducted a retrospective analysis to assess treatment outcomes utilizing PET-CT after upfront consolidative RT following immunochemotherapy containing rituximab. Through real-world data analysis, we evaluated the clinical outcomes of patients with newly diagnosed PMBCL (ND-PMBCL) treated with the standard therapeutic regimen and examined patient characteristics guiding the decision to perform or omit consolidative RT. Additionally, we analyzed the survival outcomes of subsequent treatments in patients with relapsed or refractory PMBCL (RR-PMBCL).

## Methods

### Study overview

Sixty-five patients with ND-PMBCL were identified from prospective cohort studies conducted at the Samsung Medical Center (institutional review board [IRB] number: 2022–05–078) since 2017. All the patients were pathologically diagnosed with PMBCL by two lymphoma pathologists (J.C. and Y.H.K.) based on immunohistochemistry results in accordance with the World Health Organization diagnostic criteria [14]. Patient registration for the study concluded in January 2022, and data analysis was based on information available up to June 2023, which served as the cutoff date. The IRB at the Samsung Medical Center approved this study (approval number. 2016–11–040–025). Written informed consent was obtained from each patient prior to enrollment in the study. This study was conducted in accordance with the ethical principles of the Declaration of Helsinki and the Korean Good Clinical Practice guidelines.

The following clinical and laboratory data were collected by reviewing the medical records: age; sex; Eastern cooperative oncology group performance status; presence of B symptoms; presence of extra-nodal involvement, bone marrow (BM) involvement, pleural or pericardial involvement; presence of superior vena cava (SVC) syndrome; presence of a bulky mass (≥ 10 cm); Ann Arbor stage; international prognostic index (IPI); complete blood count; lactate dehydrogenase level; C-reactive protein level; erythrocyte sedimentation rate; and β− 2 microglobulin level.

### Primary treatment

**Fig. 1:**
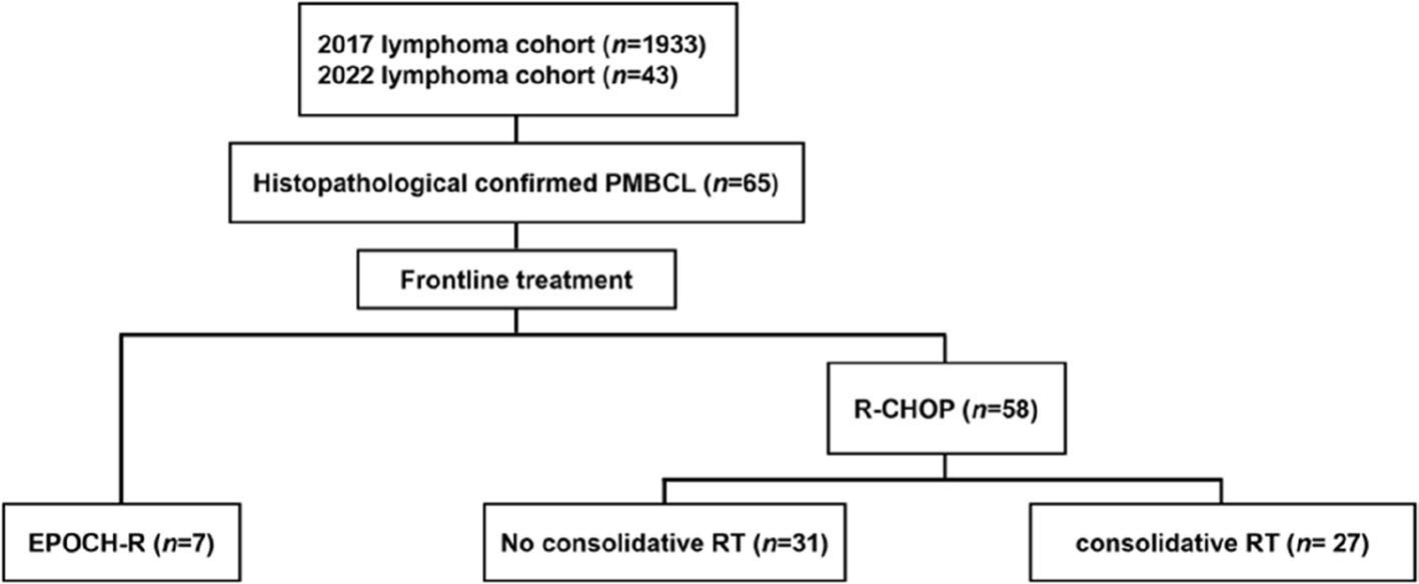
Flow diagram of the study population

The front-line immunochemotherapy regimen consisted of R-CHOP (rituximab 375 mg/m^2^, cyclophosphamide 750 mg/m^2^, doxorubicin 50 mg/m^2^, vincristine 1.4 mg/m^2^, and prednisone 40 mg/m^2^) and EPOCH-R (etoposide 50 mg/m^2^, prednisone 60 mg/m^2^, vincristine 0.4 mg/m^2^, cyclophosphamide 750 mg/m^2^, doxorubicin 10 mg/m^2^, and rituximab 375 mg/m^2^). Notably, because of reimbursement coverage from the National Health Insurance in Korea, most patients in this study (*n* = 58, 89.2%) opted for R-CHOP as their front-line regimen (Fig. 1). Treatment response was evaluated after every third cycle of front-line treatment using the Lugano classification for lymphoma, which is based on PET-CT. The degree of FDG uptake was visually quantified according to the 5-point Deauville scale (DS) [15, 16]. In this study, the PET/CT results were interpreted using the DS, with DS 1, 2, and 3 indicating negative findings and DS 4 and 5 indicating positive findings.

Following completion of the front-line treatment, consolidative RT was administered based on the joint decision of a hematologist and a radiologist. Based on the end-of-treatment (EOT) response assessment, upfront consolidative RT was recommended for patients who achieved less than a partial response (PR) and those who achieved CR but initially showed a bulky mass. The final decision for consolidative RT was taken after discussing its risks and benefits with the patient. RT was initiated 2–4 weeks following the final cycle of front-line treatment at a median total dose of 36 grays (range, 24–40 grays) delivered to the involved field at 1.8–2.0 grays/fraction, five times/week, over a period of 3–4 weeks.

### Subsequent treatment

In the case of patients where the primary treatment failed, subsequent treatment involved various systemic therapies. The choice of the subsequent therapeutic regimen was determined based on the physician's discretion and institutional protocols, and included the following options: platinum-containing chemotherapies (DHAP [dexamethasone, cytarabine, and cisplatin], ESHAP [etoposide, methylprednisolone, cytarabine, and cisplatin], GDP [gemcitabine, dexamethasone, and cisplatin], or ICE [ifosfamide, carboplatin, and etoposide] with or without rituximab), lenalidomide plus rituximab, polatuzumab plus BR (bendamustine, and rituximab), brentuximab vedotin, or immune checkpoint inhibitors (ICIs) such as pembrolizumab and nivolumab. Following salvage treatment, autologous stem cell transplantation (ASCT) was considered based on the patient's condition and disease status.

### Statistical analyses

Descriptive statistics are presented as percentages and medians. Intergroup comparison of categorical variables was conducted using the χ^2^ or Fisher's exact tests. Objective response rate (ORR) was defined as the proportion of patients who achieved a CR or PR based on their best responses obtained during treatment. CR was defined as a DS of 1, 2, or 3 at the nodal or extra-nodal sites, whereas PR was defined as a DS of 4. The Kaplan–Meier method was used to estimate overall survival (OS) and PFS. OS was assessed from the time of diagnosis to death or the last follow-up date, and PFS was estimated from the time of diagnosis to disease progression or death from any cause. To identify the factors associated with survival outcomes following PMBCL diagnosis, we performed univariate and multivariate analyses using Cox proportional hazards models. Because the variables in the univariate analysis were clinically relevant based on conventional lymphoma research, only statistically significant variables were included in the multivariate model. All data were analyzed using Statistical Package for Social Sciences software (version 24.0; IBM Corp., Armonk, NY, USA).

## Results

### Patient characteristics

The baseline demographics of 65 patients with ND-PMBCL at diagnosis included in the study are presented in Table 1. The median patient age was 32 years (range, 16–86 years). Fifty-two patients (80.0%) were diagnosed with PMBCL before 40 years of age. The cohort had more females (*n* = 35, 58.5%) than males (*n* = 27, 41.5%). At diagnosis, 35% of patients (*n* = 23) showed extra-nodal lymphoma involvement, whereas BM infiltration was infrequent (*n* = 2, 3.1%). PMBCL invaded the pleura (*n* = 7, 10.8%) or pericardium (*n* = 9, 13.8%) in some cases, whereas SVC syndrome with a bulky mass was observed in 10.8% (*n* = 7) of patients. More than half the patients (*n* = 34, 52.3%) included in this study presented with advanced disease (stage III or IV). Based on IPI, approximately 85% of the patients (*n* = 55) were classified as having a low or low/intermediate risk.

**Table 1:** Baseline demographics and disease characteristics at diagnosis

Characteristics		Total (*n* = 65)	R-EPOCH (*n* = 7)	R-CHOP (*n* = 31)	R-CHOP + RT (*n* = 27)	*P-value
Age (years)	Median (range)	32 (16–86)	32 (19–39)	32 (20–86)	31.5 (16–57)	
	≤ 40	52 (80.0%)	7 (100.0%)	21 (67.7%)	24 (88.9%)	0.054
	> 40	13 (20.0%)	0 (0.0%)	10 (32.3%)	3 (10.3%)	
Sex	Male	27 (41.5%)	3 (42.9%)	13 (41.9%)	11 (40.7%)	0.927
	Female	38 (58.5%)	4 (57.1%)	18 (58.1%)	16 (59.2%)	
ECOG-PS	0–1	54 (83.1%)	6 (85.7%)	25 (80.6%)	23 (85.2%)	0.648
	2–4	11 (16.9%)	1 (14.3%)	6 (19.4%)	4 (14.8%)	
B symptom	Present	2 (3.1%)	1 (14.3%)	1 (3.2%)	0 (0.0%)	0.346
LDH	Elevated	56 (86.2%)	7 (100.0%)	25 (80.6%)	24 (88.9%)	0.387
β2-microglobulin	Elevated	4 (6.2%)	1 (14.3%)	1 (3.2%)	2 (7.4%)	0.460
CRP	Elevated	22 (33.8%)	4 (57.1%)	6 (19.3%)	12 (44.4%)	0.034
ESR	Elevated	38 (58.5%)	1 (14.3%)	22 (70.9%)	15 (55.6%)	0.085
Extra-nodal involvement	Absence	42 (64.6%)	1 (14.3%)	25 (80.6%)	16 (59.2%)	0.200
	1 site	12 (18.5%)	3 (42.9%)	3 (9.7%)	6 (22.2%)	
	≥ 2 sites	11 (16.9%)	3 (42.9%)	3 (9.7%)	5 (18.5%)	
Bone marrow involvement	Present	2 (3.1%)	0 (0.0%)	1 (3.2%)	1 (3.7%)	0.921
Pleural involvement	Present	7 (10.8%)	1 (14.3%)	3 (9.7%)	3 (10.3%)	0.858
Pericardial involvement	Present	9 (13.8%)	2 (28.6%)	3 (9.7%)	4 (14.8%)	0.549
SVC syndrome	Present	7 (10.8%)	0 (0.0%)	3 (9.7%)	4 (14.8%)	0.549
CD30 expression	Present	51 (78.5%)	6 (85.7%)	22 (70.9%)	23 (85.2%)	0.195
Bulky (≥ 10 cm) mass	Present	8 (12.3%)	0 (0.0%)	4 (12.9%)	4 (14.8%)	0.833
Ann Arbor stage	I/II	31 (47.7%)	1 (14.3%)	17 (54.8%)	13 (48.1%)	0.611
	III/IV	34 (52.3%)	6 (85.7%)	14 (45.2%)	14 (51.9%)	
IPI	Low	34 (52.3%)	1 (14.3%)	18 (58.1%)	15 (55.6%)	0.206
	Low/Intermediate	21 (32.3%)	3 (42.9%)	11 (35.5%)	7 (25.9%)	
	High/Intermediate	9 (13.8%)	3 (42.9%)	1 (3.2%)	5 (18.5%)	
	High	1 (1.5%)	0 (0.0%)	1 (3.2%)	0 (0.0%)	

*Abbreviations: R-CHOP* rituximab, cyclophosphamide, doxorubicin, vincristine, and prednisone, *RT* radiation therapy, *R-EPOCH* rituximab, etoposide, cyclophosphamide, doxorubicin, vincristine, and prednisone, *ECOG-PS* Eastern cooperative oncology group performance status, *LDH* lactate dehydrogenase, *CRP* C-reactive protein; ESR, erythrocyte sedimentation rate, *SVC* superior vena cava, *IPI* international prognostic index

^*^ Comparison between the R-CHOP and R-CHOP + RT groups

The patients were divided into three groups based on their primary treatment: (1) EPOCH-R (EPOCH-R group*, n* = 7, 10.7%), (2) R-CHOP only (R-CHOP group, *n* = 31, 47.7%), and (3) R-CHOP with consolidative RT (R-CHOP + RT group, *n* = 27, 41.5%) (Fig. 1). Patients in the EPOCH-R group did not receive consolidative RT. The median number of front-line immunochemotherapy cycles administered to patients in each group was 6 (range, 1–6), 6 (range, 3–6), and 6 (range, 6–8), respectively. Patients with more unfavorable baseline characteristics tended to receive the EPOCH-R regimen, although the difference was not significant (Table 1).

### Primary treatment for ND-PMBCL

**Fig. 2:**
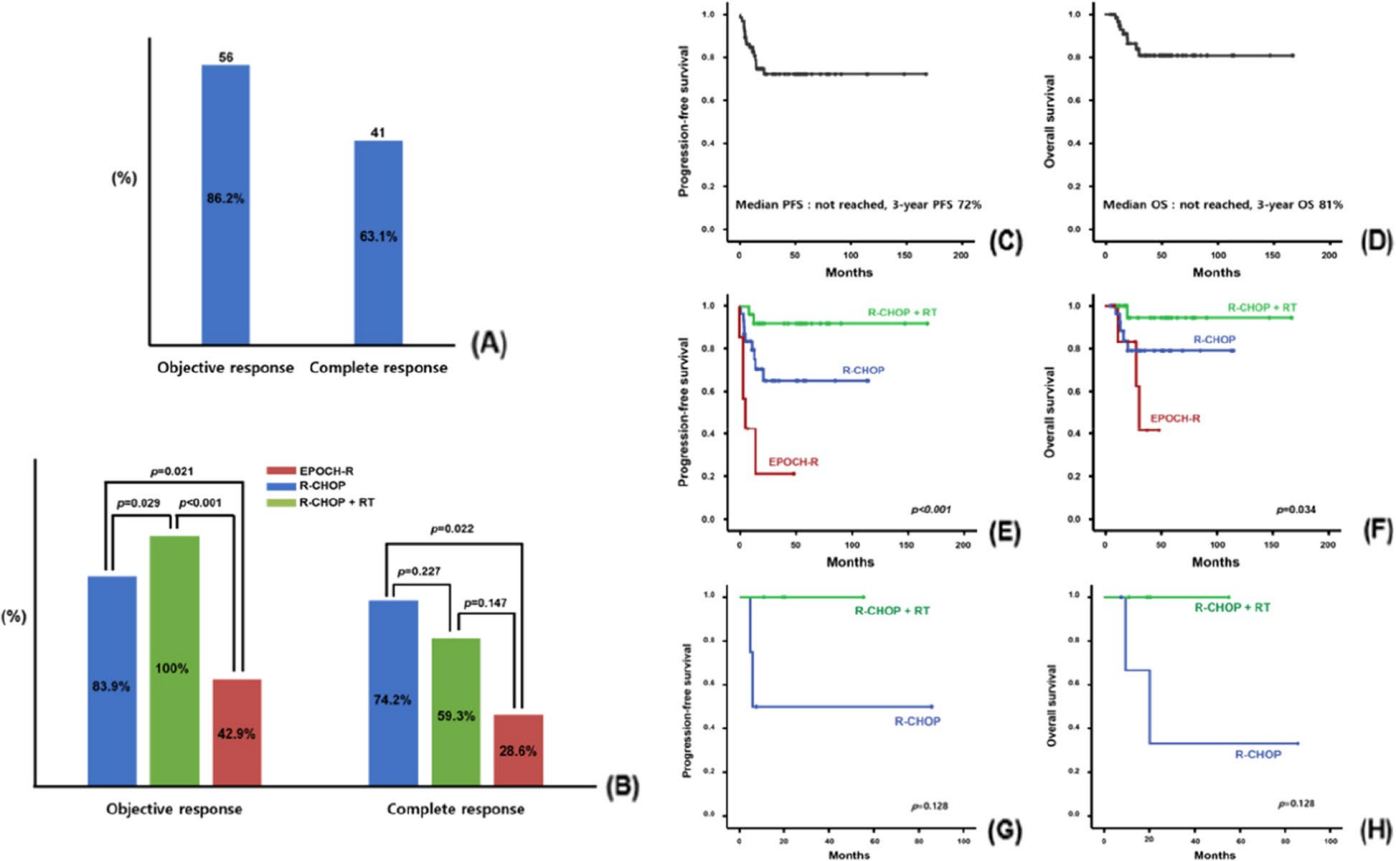
Overall achievement of an objective response and/or complete response (**A**). Comparison of responses to the three primary treatment regimens (**B**). Kaplan–Meier curves of progression-free survival (PFS) (**C**) and overall survival (OS) (**D**) according to primary treatment. Comparison of PFS (**E**) and OS (**F**) according to the three primary treatment regimens. Comparison of PFS (**G**) and OS (**H**) according to radiation therapy in patients with bulky disease at diagnosis

Based on the best response, the estimated OR and CR rates for all patients were 86.2% (*n* = 56) and 63.1% (*n* = 41), respectively (Fig. 2A). The EPOCH-R group achieved an ORR of 42.9% (3/7) and CR rate of 28.6% (2/7). The R-CHOP group achieved an ORR of 83.9% (26/31) and a CR rate of 74.2% (23/31). All patients in the R-CHOP + RT group achieved an objective response (*n* = 27, 100%), whereas the CR rate was 59.3% (16/27). The ORRs were significantly different among the three primary treatment groups (*p* < 0.001), whereas no significant differences were noted in the CR rates among the groups (*p* = 0.067) (Fig. 2B).

With a median follow-up duration of 35.7 months, the PFS and OS did not reach the median value, and the estimated median 3-year PFS and OS for primary treatment were 72% and 81%, respectively (Fig. 2C, D). In the PFS analysis, survival curves according to the primary treatment were clearly distinguishable, with the EPOCH-R group (6.0 months; 95% confidence interval [CI], 0.868–11.132) showing a significantly worse outcome than the R-CHOP (median survival not reached [NR]) and R-CHOP + RT groups (median survival NR) (Fig. 2E). The R-CHOP + RT group (median survival NR) had a more favorable median OS than the R-CHOP (median survival NR) and EPOCH-R groups (30.5 months; 95% CI, 25.284–35.716) (Fig. 2F). In particular, patients with bulky disease at diagnosis (*n* = 8) showed a trend toward improved survival when administered with consolidative RT, although the difference was not significant (Fig. 2G, H).

We performed Cox regression analysis to identify factors that predict better survival outcomes following primary treatment. The inclusion of consolidative RT in the primary treatment (hazard ratio [HR] 0.81; 95% CI, 0.016–0.411; *p* = 0.002) and negative EOT PET-CT findings (HR 9.843; 95% CI, 1.414–68.496; *p* = 0.021) were associated with improved PFS. However, no clinical factor was identified that significantly affected the OS (Table 2).

**Table 2:** Univariate and multivariate analyses of progression-free survival and overall survival

Variables	Progression-free survival			
	**Univariate**		**Multivariate**	
	**HR (95%CI)**	***P-value***	**HR (95%CI)**	***P-value***
Age > 40 years	1.291 (0.342–4.878)	0.706	-	-
Male	1.220 (0.372–3.998)	0.743	-	-
ECOG-PS ≥ 2	0.036 (0.000–24.239)	0.317	-	-
Stage ≥ III	2.950 (0.782–11.126)	0.110	-	-
IPI ≥ 3	1.532 (0.331–7.094)	0.586	-	-
Bulky (≥ 10 cm) disease	1.740 (0374–8.103)	0.480	-	-
LDH elevated	0.829 (0.179–3.847)	0.811	-	-
CRP elevated	1.246 (0.453–3.430)	0.671		
ESR elevated	0.425 (0.157–1.152)	0.093		
Extra-nodal involvement	1.343 (0.393–4.591)	0.638	-	-
Pleural involvement	1.530 (0.329–7.110)	0.587	-	-
Pericardial involvement	0.041 (0.000–143.900)	0.443	-	-
SVC syndrome present	0.041 (0.000–105.680)	0.425	-	-
Upfront radiation therapy	**0.210 (0.045–0.973)**	**0.046**	**0.81 (0.016–0.411)**	**0.002**
Positive Interim PET-CT	**6.455 (1.709–24.383)**	**0.006**	2.393 (0.327–17.532)	0.391
Positive EOT PET-CT	**7.858 (2.279–27.098)**	**0.001**	**9.843 (1.414–68.496)**	**0.021**
**Variables**	**Overall survival**			
	**Univariate**		**Multivariate**	
	**HR (95%CI)**	***P-value***	**HR (95%CI)**	***P-value***
Age > 40 years	0.632 (0.074–5.410)	0.675	-	-
Male	1.362 (0.275–6.748)	0.705	-	-
ECOG-PS ≥ 2	0.037 (0.000–389.171)	0.486	-	-
Stage ≥ III	5.744 (0.670–49.227)	0.111	-	-
IPI ≥ 3	1.698 (0.198–14.603)	0.629	-	-
Bulky (≥ 10 cm) disease	3.855 (0.705–21.066)	0.119	-	-
LDH elevated	1.278 (0.149–10.969)	0.823	-	-
CRP elevated	0.961 (0.240–3.845)	0.955		
ESR elevated	0.253 (0.063–1.020)	0.053		
Extra-nodal involvement	2.729 (0.549–13.570)	0.220	-	-
Pleural involvement	1.425 (0.166–12.226)	0.747	-	-
Pericardial involvement	0.043 (0.000–190.575)	0.635	-	-
SVC syndrome present	0.042 (0.000–354.308)	0.583	-	-
Upfront radiation therapy	0.200 (0.023–1.711)	0.142	-	-
Positive Interim PET-CT	20.712 (0.085–488.187)	0.181		
Positive EOT PET-CT	71.273 (0.007–901.532)	0.264	-	-

*Abbreviations: HR*, hazard ratio, *CI* confidence interval, *ECOG-PS* Eastern cooperative oncology group performance status; IPI, International Prognostic Index; LDH, lactate dehydrogenase; *CRP* C-reactive protein; ESR erythrocyte sedimentation rate, SVC superior vena cava, *PET-CT* positron emission tomography-computed tomography, *EOT* end-of-treatment

### Survival outcomes according to response evaluation with PET-CT

**Fig. 3:**
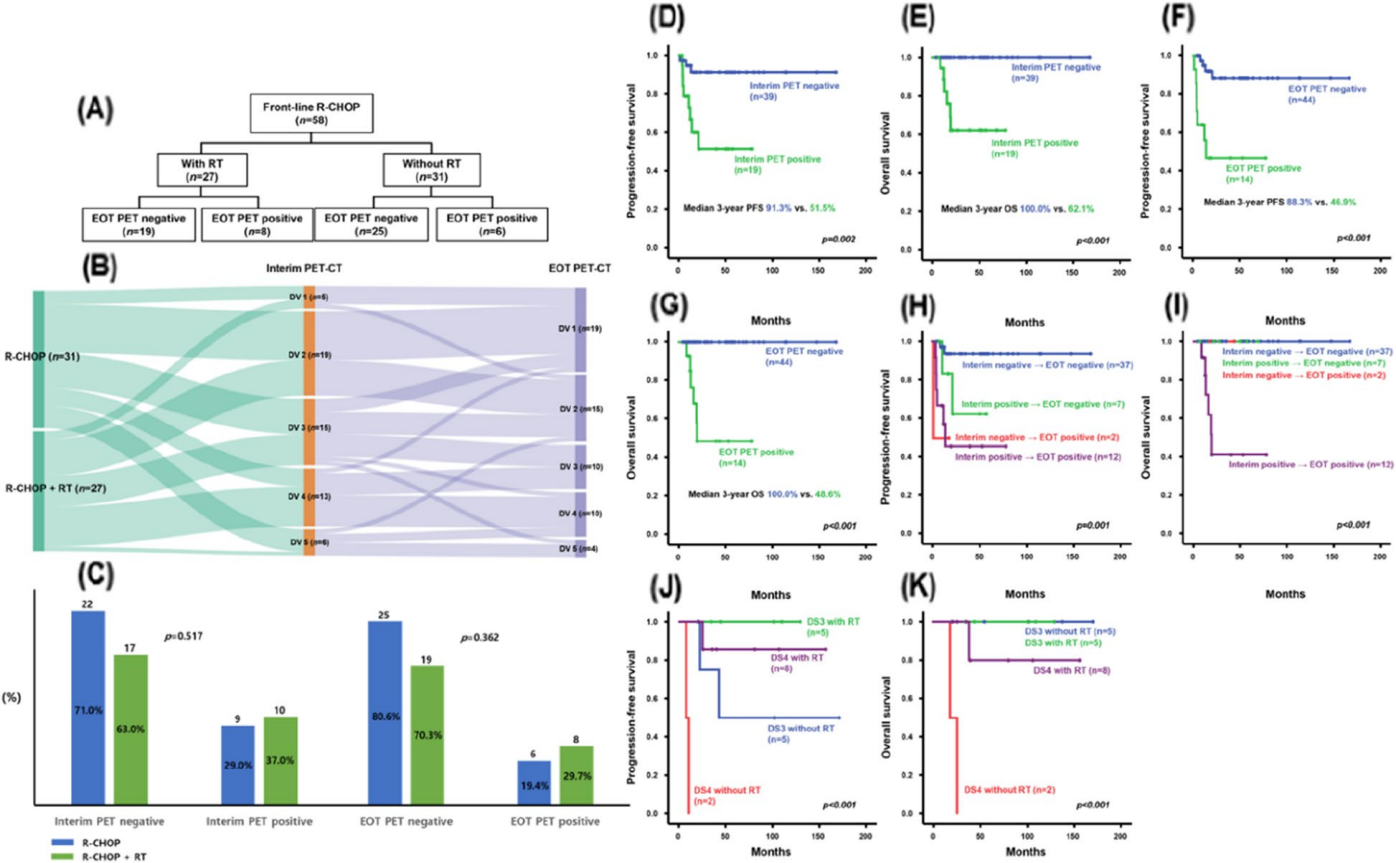
End-of-treatment (EOT) PET-CT) results according to radiation therapy (RT) in patients treated with R-CHOP (**A**). Sankey diagram of patients treated with R-CHOP showing interim and EOT PET-CT responses (**B**). Comparison of interim and EOT PET-CT responses according to RT in patients treated with R-CHOP (**C**). Comparison of progression-free survival (PFS) (**D**) and overall survival (OS) (**E**) according to interim PET-CT findings. Comparison of PFS (**F**) and OS (**G**) according to EOT PET-CT findings. Comparison of PFS (**H**) and OS (**I**) according to the interim and EOT PET-CT findings. Comparison of PFS (**J**) and OS (**K**) according to RT administration in patients with DS 3 and 4. PET-CT; positron emission tomography-computed tomography, R-CHOP; rituximab, cyclophosphamide, doxorubicin, vincristine, and prednisone, DS; Deauville scale

Of the 58 patients uniformly treated with front-line R-CHOP (Fig. 3A), 39 (67.2%) exhibited negative PET-CT findings (DS 1–3), whereas the remaining 19 (32.8%) did not (DS 4 or 5) during the interim evaluation. After completing the front-line treatment, 44 patients (75.9%) achieved negative PET/CT results (Fig. 3B). Most patients (37/39, 94.9%) who had negative PET-CT findings in the interim evaluation maintained PET-CT negativity during the EOT assessment. In contrast, approximately 37% (7/19) of patients with positive PET-CT results in the interim evaluation acquired PET-CT negativity in the EOT assessment (Fig. 3B). During the interim evaluation, 71% (22/31) and 63% (17/27) of the patients in the R-CHOP group and R-CHOP + RT groups, respectively, achieved PET-CT negativity. At the EOT evaluation, 80.6% (25/31) and 70.3% (19/27) of the patients in the R-CHOP and R-CHOP + RT groups, respectively, presented with negative PET-CT findings. No significant differences were observed in the interim (*p* = 0.517) or EOT PET-CT (*p* = 0.362) results between the R-CHOP and R-CHOP + RT groups (Fig. 3C).

Achievement of negative interim PET/CT findings (*n* = 39) correlated with significantly better survival outcomes, whereas failure to achieve negative PET/CT findings (*n* = 19) during the interim evaluation was associated with suboptimal survival outcomes (Fig. 3D, E). Additionally, patients who failed to achieve EOT PET-CT negativity (*n* = 14) had significantly worse survival outcomes regardless of interim PET-CT results. In contrast, among those who achieved EOT PET-CT negativity (*n* = 44), the interim PET-CT findings (although not significant) showed a trend toward influencing survival outcomes (*p* = 0.058). Notably, patients who remained PET/CT-positive at both the interim and EOT evaluations experienced rapid disease progression and higher mortality (Fig. 3F-I). Logistic regression analysis revealed that positive interim PET-CT findings (*p* < 0.001) were significantly associated with positive EOT PET-CT (Table 3).

**Table 3:** Logistic regression analysis of factors associated with EOT PET-CT results

Variables	Overall survival			
	**Univariate**		**Multivariate**	
	**HR (95%CI)**	***P-value***	**HR (95%CI)**	***P-value***
Age > 40 years	0.927 (0.216–3.986)	0.919	-	-
Male	1.588 (0.473–5.330)	0.454	-	-
ECOG-PS ≥ 2	2.533 (0.598–10.738)	0.207	-	-
Stage ≥ III	0.750 (0.223–2.521)	0.642	-	-
IPI ≥ 3	1.300 (0.223–7.578)	0.771	-	-
Bulky (≥ 10 cm) disease	**4.000 (0.849–18.836)**	**0.080**	2.018 (0.273–14.949)	0.492
LDH elevated	0.579 (0.124–2.700)	0.487	-	-
Extra-nodal involvement	1.481 (0.412–5.322)	0547	-	-
Pleural involvement	1.667 (0.271–10.244)	0.581	-	-
Pericardial involvement	1.300 (0.223–7.578)	0.771	-	-
SVC syndrome present	1.300 (0.223–7.578)	0.771	-	-
Positive interim PET-CT	**31.714 (5.788–173.779)**	** < 0.001**	**28.755 (5.166–160.038)**	** < 0.001**

*Abbreviations: EOT* end-of-treatment, *PET-CT* positron emission tomography-computed tomography, *HR* hazard ratio, *CI* confidence interval, *ECOG-PS* Eastern cooperative oncology group performance status, *IPI* international prognostic index, *LDH* lactate dehydrogenase, *SVC* superior vena cava

We were particularly interested in determining the role of consolidative RT based on the EOT PET/CT results to further define the indications for upfront consolidative RT in patients treated with front-line R-CHOP. Patients who achieved a DS of 1 (*n* = 19) or 2 (*n* = 15) on EOT PET/CT showed no difference in survival outcomes with the administration of consolidative RT, as both the RT and non-RT groups demonstrated a median 3-year PFS of 92.9% and an OS of 100%. In patients with a DS of 3 (*n* = 10), which corresponded to negative PET-CT findings, administration of consolidative RT tended to prolong PFS but did not result in any difference in OS. However, in those with a DS score of 4, consolidative RT had a significant effect on both PFS (*p* = 0.001) and OS (*p* = 0.001) (Fig. 3J, K).

### RR-PMBCL after primary treatment failure

**Fig. 4:**
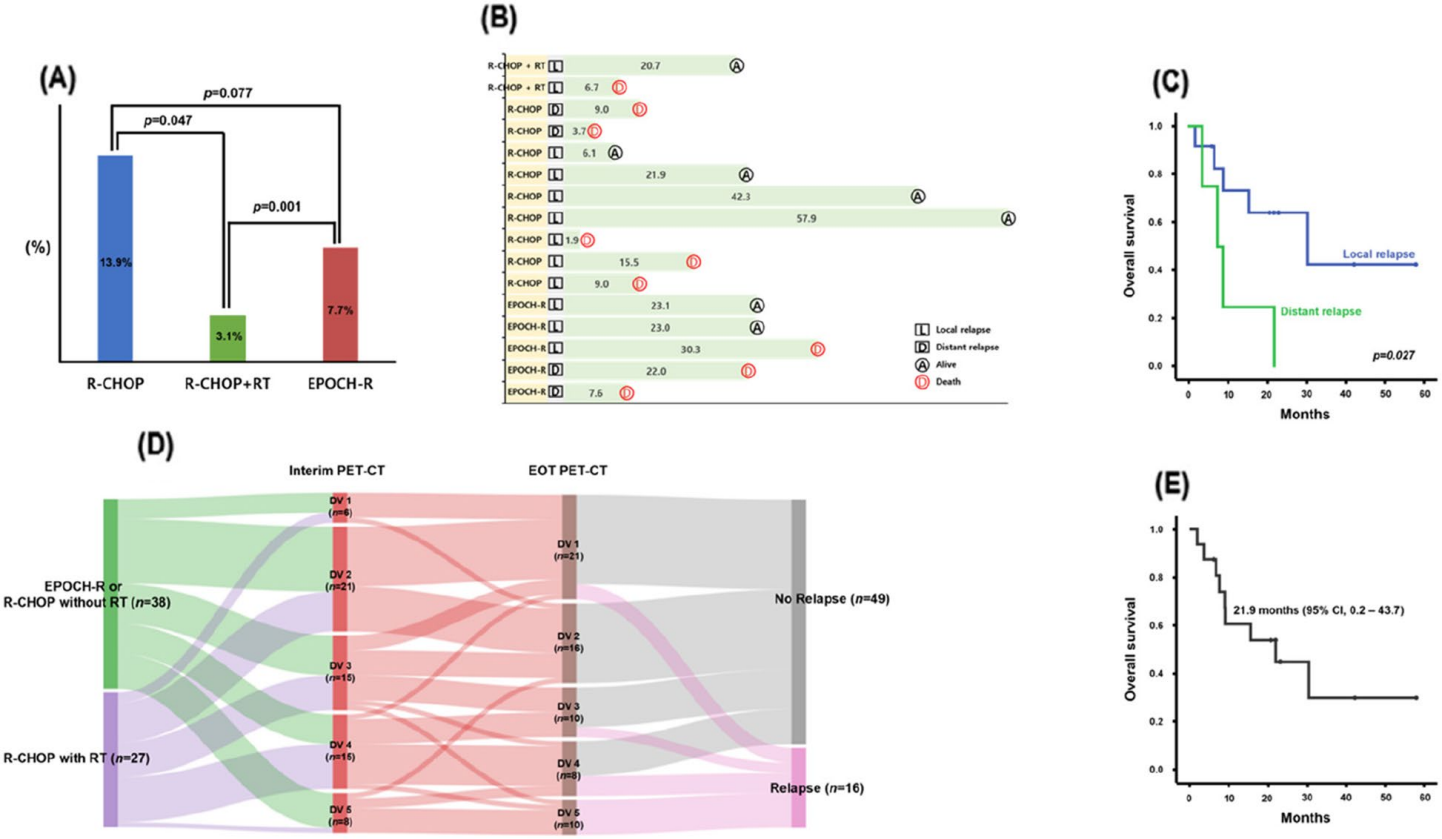
Comparison of disease relapse among the three primary treatment modality groups (**A**). Swimmer plot of relapsed patients (**B**). Comparison of overall survival (OS) according to patterns of disease relapse (**C**). Sankey diagram showing the PET-CT response and disease relapse (**D**). Kaplan–Meier curves of OS of patients with disease relapse (**E**). PET-CT; positron emission tomography-computed tomography

Sixteen patients (24.6%) relapsed after primary treatment and 15 (93.8%) presented with early RR-PMBCL, defined as relapse within 12 months of treatment completion. Disease relapse occurred in five (7.7%), nine (13.8%), and two (3.1%) patients in the EPOCH-R, R-CHOP, and R-CHOP + RT groups, respectively (*p* = 0.002), with the R-CHOP + RT group exhibiting a relatively lower relapse rate than the other groups (Fig. 4A). Eleven (68.8%) patients experienced local relapse and five (31.2%) distant relapse (Fig. 4B), with no observed differences in the relapse type based on primary treatment (*p* = 0.571). Disease relapse adjacent to the primary site was associated with better survival outcomes than disease relapse at distant sites (Fig. 4C). Approximately 60% (11/18) of the patients with positive EOT PET-CT showed disease relapse, whereas only 10% (5/47) of the patients with negative EOT PET-CT showed disease relapse (Fig. 4D). The estimated median OS for RR-PMBCL was 21.9 months (95% CI, 0.2–43.7 months) (Fig. 4E).

**Fig. 5:**
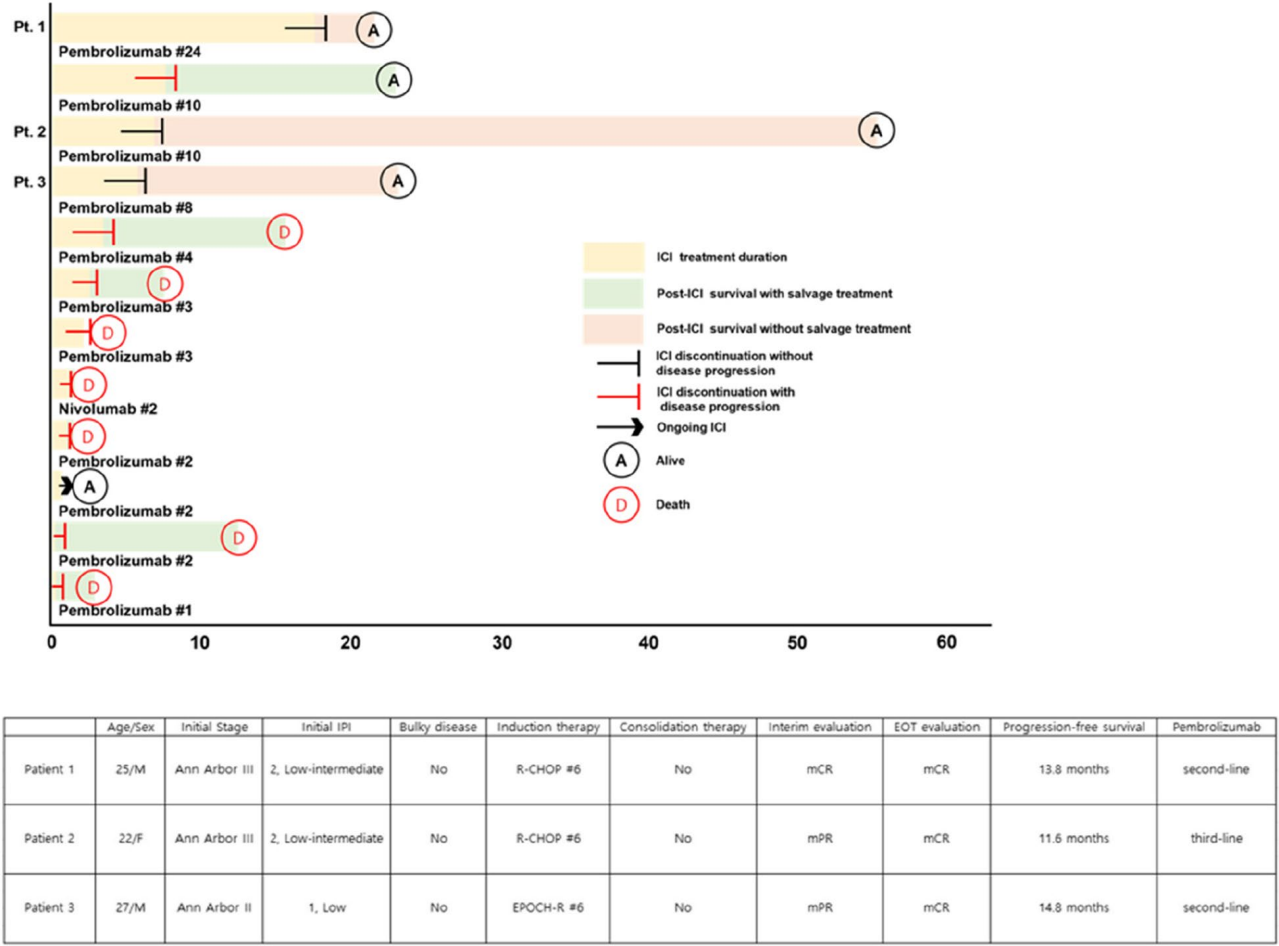
Swimmer plot of relapsed patients treated with immune checkpoint inhibitors

All patients who relapsed received various subsequent treatments. Owing to the small number of patients assigned to each salvage treatment, comparison of the efficacy of these treatments remained challenging. In this study, a notable proportion (75.0%, 12/16) of patients received ICIs, including pembrolizumab (*n* = 11) and nivolumab (*n* = 1). Prior to receiving ICI therapy, the patients underwent a median of three (range, 1–6) lines of treatment. The median number of cycles of ICI therapy was three (range, 1–24), with a median treatment duration of 2.2 months. The ORR of ICI therapy was 58.3% (*n* = 7), and a quarter of the patients (*n* = 3) achieved CR. Two patients (patients 1 and 2) who achieved CR discontinued treatment after seven months and two years of pembrolizumab therapy, respectively. Another patient (patient 3) underwent consolidative ASCT after achieving CR with six months of pembrolizumab therapy. These three patients remained alive without disease progression at the cutoff date (Fig. 5). Despite ICI therapy, two-thirds of patients (66.6%, 8/12) eventually experienced disease progression. The 1-year PFS and OS rates after ICI therapy were 20% and 54%, respectively. No instances of treatment discontinuation, dose reduction, or mortality due to adverse effects were observed during ICI therapy.

## Discussion

Several previous studies on PMBCL have consistently demonstrated the efficacy of rituximab-containing immunochemotherapy; however, owing to heterogeneous front-line regimens and RT indications, as well as varying protocols, a uniform conclusion regarding the role of consolidative RT has not been reached (Table 4). In this study, we evaluated the role of upfront consolidative RT following front-line immunochemotherapy in patients with ND-PMBCL. The integration of consolidative RT into primary treatment provided clinical benefits with limited adverse events in this study, which may be attributed to the short follow-up duration. Patients who received R-CHOP as a front-line treatment and underwent consolidative RT had prolonged PFS and a potentially reduced risk of disease relapse. Although a favorable trend was observed, it did not translate into a significant extension of OS, possibly because of the small sample size and limited follow-up duration.

**Table 4:** Summary of studies on rituximab-containing treatment in primary mediastinal large B-cell lymphoma

Reference (year)	Study size	Study type	Front-line regimen	Consolidation	Median age, years	PFS/EFS (median)	OS (median)	Prognostic factors
HK Ahn (2010)	35	Retrospective	R-CHOP (60%) CHOP (40%)	RT (57.1%)	30 (range, 17–79)	2 yr PFS 79.0% vs. 50.0%	2 yr OS 82.7% vs. 57.1%	Achievement of CR (OS, PFS)
Rieger (2011) [6]	87	Prospective	R + CHOP-like (50.6%) CHOP-like (49.4%)	RT (70.1%)	36 (range, 27–43)	3 yr EFS 78% vs. 52%	3 yr OS 89% vs. 78%	Rituximab-containing regimen (EFS) age-adjusted IPI (EFS) bulky disease (OS, EFS)
Vassilakopoulos (2012)	76	Retrospective	R-CHOP (100%)	RT (68.4%)	31.5 (range, 17–73)	5 yr EFS 80%	5 yr OS 89%	None
Xu (2013)	79	Retrospective	R-CHOP (49.4%) CHOP-like (50.6%)	RT (75.9%)	29 (range, 13–54)	5 yr PFS 76.7% vs. 44.2%	5 yr OS 83.7% vs. 48.3%	Rituximab-containing regimen (OS, PFS) RT (PFS) LDH (OS, PFS) stage III/IV (OS, PFS)
Dunleavy (2013) [12]	51	Phase 2	DA-EPOCH-R (100%)	None	30 (range, 19–52)	5 yr PFS 93%	5 yr OS 97%	N/A
Aoki (2014)	345	Retrospective	R-CHOP (54.2%) DA-EPOCH-R (2.6%) CHOP (12.7%)	RT (42%) ASCT (16.5%)	32 (range, 17–83)	4 yr PFS 75% vs. 54%	4 yr OS 91% vs. 77%	Pleural or pericardial effusion (PFS) stage III/IV (PFS)
Soumerai (2014)	63	Retrospective	R-CHOP (100%)	RT (77%)	37 (range, 20–82)	5 yr PFS 68^	5 yr OS 79%	Advanced stage (PFS) age > 60 years (OS, PFS) multiple extra-nodal site (OS, PFS)
HJ Ahn (2014)	25	Retrospective	R-CHOP (64.0%) CHOP (28.0%) Others (8.0%)	RT (36%) ASCT (16%)	31 (range, 15–85)	3 yr PFS 71.4% vs. 68.2%	3 yr OS 85.7% vs. 62.5%	Poor performance status (OS) B symptoms (OS) SVC syndrome (OS)
Gleeson (2016)	50	Phase 3 Subgroup analysis	R-CHOP (100%)	RT (58%)	38.5 (range, 22–78)	5 yr PFS 79.8%	5 yr OS 83.8%	N/A
Lisenko (2017) [7]	80	Retrospective	R-CHO(E)P (56%) CHO(E)P (44%)	RT (88%) ASCT (30%)	37 (range, 18–64)	10 yr PFS 95% vs. 67%	10 yr OS 92% vs. 72%	Rituximab-containing regimen (OS, PFS) Achievement of CR (OS, PFS)
Giulino-Roth (2017)	118	Retrospective	DA-EPOCH-R (100%)	RT (16.1%)	34 (range, 21–70)	3 yr EFS 87.4%	3 yr OS 97.1%	Negative end-of-therapy PET (EFS)
Shah (2018) [19]	132	Retrospective	R-CHOP (54.2%) DA-EPOCH-R (2.6%)	RT (32.6%)	35 (range, 18–77)	2 yr PFS 76% vs. 85%	2 yr OS 89% vs. 91%	N/A
Melani (2018)	93	Retrospective	DA-EPOCH-R (100%)	None	31 (range, 18–68)	8 yr EFS 90.6%	8 yr OS 94.7%	N/A
Messmer (2019)	43	Retrospective	R-CHOP (76.7%) DA-EPOCH-R (21%)	RT (23%)	36 (range, 16–60)	3 yr PFS 93% vs. N/A	3 yr OS 100% vs. N/A	N/A
Chan (2019) [18]	124	Retrospective	R-CHOP (62.9%) DA-EPOCH-R (37.1%)	RT (29.8%)	27 (range, 11–72)	5 yr PFS 56.5% vs. 90%	5 yr OS 76.1% vs. 96.9%	B symptoms (OS) Primary treatment regimen (PFS)
Malenda (2020)	53	Retrospective	R-CHOP (47.6%) DA-EPOCH-R (52.4%)	RT (49.1%) ASCT (18.9%)	30 (range, 18–80)	1 yr PFS 87% vs. 73.9%	1 yr OS 100% vs. 92%	ASCT (OS)
Zhou (2020)	166	Retrospective	R-CHO(E)P (86.7%) R-HyperCVAD (11.4%) others (1.8%)	RT (51.2%) ASCT (15.1%)	33 (range, 11–64)	5 yr PFS 70%	5 yr OS 79%	Ki- 67 ≥ 70% (OS, PFS) MUM1 status (OS) CBC lymphocyte/monocyte ratio (PFS)
Hayden (2020) [24]	159	Retrospective	R-CHOP (94%) R-containing (6%)	RT (44%)	36 (range, 19–84)	5 yr PFS 79%	5 yr OS 89%	Stage III/IV (PFS) Pleuralpericardial effusion (PFS)
Stepanishyna (2020)	69	Prospective (Abstract)	R-CHOP (47.8%) DA-EPOCH-R (52.2%)	RT (N/A)	27 (range, 17–45)	5 yr PFS 64.1% vs. 90.9%	5 yr OS 73.8% vs. 97.7%	N/A
Wästerlid (2021)	172	Prospective	R-CHO(E)P (73.8%) DA-EPOCH-R (7%) R-containing (11%)	RT (17%)	37.5 (range, 18–85)	5 yr relative survival 88%		Increasing age (relative survival) Performance status (relative survival) age-adjusted IPI (relative survival)
Held (2023)	131	Phase 3 subgroup analysis	R-CHOP (100%)	RT (62.6%)	34 (range, 18–60)	3 yr PFS 93%	3 yr OS 97%	LDH (OS, PFS) stage III/IV (OS, PFS)
Zucca (2023) [17]	545	Phase 3 (Abstract)	R-containing (100%)	RT (25%)	N/A	30 month PFS 98.5% vs. 96.2%	5 yr OS 99% vs. 99%	N/A
This study (2024)	65	Retrospective	R-CHOP (90%) DA-EPOCH-R (10%)	RT (44.6%)	32 (16–86)	3 yr PFS 72%	3 yr OS 81%	Consolidative RT (PFS) End-of-therapy PET (PFS)

*Abbreviations: PFS* progression-free survival, *EFS* event-free survival, *OS* overall survival, *RT* radiation therapy, *ASCT*, autologous stem cell transplant; *R* rituximab, *CHOP*, cyclophosphamide, doxorubicin, vincristine, prednisolone, *DA-EPOCH* dose-adjusted rituximab, etoposide, cyclophosphamide, doxorubicin, vincristine, and prednisone, *CHO(E)P* cyclophosphamide, doxorubicin, vincristine, etoposide, prednisolone, *CR* complete response, *IPI* international prognostic index, *LDH* lactate dehydrogenase, *PET* positron-emission tomography, *N/A* not available, *SVC* superior vena cava

As demonstrated by a recent large prospective study [17], the omission of consolidative RT in patients with PMBCL is advocated particularly in patients who have received intensive front-line immunochemotherapy or have attained a complete metabolic response in EOT PET-CT. In support of this, we found that consolidative RT did not yield significant benefits in patients with DS of 1 or 2 on EOT PET-CT. Among patients with a DS of 3, consolidative RT was associated with improved survival, although this association was not significant. Conversely, RT provided significant survival benefits in patients with a DS of 4. (Fig. 3J, K). Patients who received consolidative RT had lower rates of both local and distant relapses than those who did not (Fig. 4A, B). This significant reduction in the ORR appears to have led to an increase in the PFS. In previous studies, consolidative RT demonstrated sufficient survival benefits along with manageable toxicity [8, 9]. Although the recently introduced intensified front-line immunochemotherapy regimen has diluted the role of consolidative RT, it remains a valid option for patients meeting specific criteria. Data from Asian studies support the implementation of consolidative RT following R-CHOP in patients with ND-PMBCL who present with bulky disease [18]. Despite the limitation of small sample size (*n* = 8), our study also demonstrated that patients with initial bulky disease tended to have better survival when receiving consolidative RT than those who did not. Consequently, we advocate the use of consolidative RT in patients receiving front-line R-CHOP who have remnant lesions indicating a DS of 4 on EOT PET-CT or initial bulky disease.

Three primary treatment strategies incorporating standard immunochemotherapy regimens were evaluated: EPOCH-R, R-CHOP only, and R-CHOP + RT. At first glance, the clinical outcomes of EPOCH-R appeared somewhat disappointing compared to those of the other two treatments; however, a direct comparison of the efficacy of the front-line regimen was challenging for several reasons. First, the limited number of patients (*n* = 7, 10.8%) treated with EPOCH-R hindered a comprehensive evaluation. Second, there was significant heterogeneity in the baseline characteristics of the patients, with a notable tendency for those with a deteriorated disease status to be treated with EPOCH-R (Table 1). According to previous studies (Table 4), favorable outcomes have been reported for EPOCH-R as intensive chemotherapy and mediastinal RT can be safely omitted when using this treatment. However, treatment-related hematologic toxicities are prevalent in patients treated with EPOCH-R. Thus, relatively older and frail patients with PMBCL may not be suitable candidates for intensive immunochemotherapy as the primary treatment. In a comparative study of R-CHOP and dose-adjusted R-EPOCH (DA-R-EPOCH) as front-line management strategies for PMBCL [19], patients receiving DA-R-EPOCH had higher CR rates (84% vs. 70%, *p* = 0.046), but were more likely to experience treatment-related toxicities. Therefore, R-CHOP may be an excellent alternative for patients with PMBCL who are frail and are expected to have poor performance.

As in other types of lymphomas, FDG PET-CT is a useful tool for evaluating treatment response in PMBCL. However, post-treatment remnant inflammatory tissue may lead to false-positive PET/CT findings [12], which can negatively affect its excellent negative predictive value [17]. We reaffirmed that PET-CT is a suitable tool for assessing treatment response in PMBCL; in particular, survival outcomes were better when PET-CT negativity was achieved early (Fig. 3D-G). Additionally, PET-CT assisted in identifying patients who would benefit from consolidative RT after primary treatment (Fig. 3J, K). Recently, to overcome the limitations of the 5-point DS based on the visual interpretation of FDG uptake, quantitative evaluation methods using total lesion glycolysis have been introduced that have better predictive value [20, 21]. Moreover, monitoring circulating tumor DNA can enhance the evaluation of therapeutic response, allow accurate identification of false-positive PET-CT results caused by residual mediastinal uptake, and help identify patients who may benefit from consolidative RT [22, 23]. Advancements in EOT evaluation modalities are expected to clarify the role and indications of consolidative RT in PMBCL in the near future.

Although patients with PMBCL generally have better survival outcomes than those with conventional DLBCL, treatment failure is common and often occurs soon after the completion of primary treatment. In this study, approximately a quarter of the patients experienced primary treatment failure, with the majority experiencing early relapse. Local disease relapse was more common than distant relapse (Fig. 4B), and patients with local relapse showed better survival outcomes (Fig. 4C). In cases of RR-PMBCL, approximately 30% of patients achieved a long-term response to various salvage therapies, which is consistent with findings from other studies. Systemic treatment followed by high-dose chemotherapy and ASCT remains the mainstay of salvage treatment. However, although these treatment strategies are particularly beneficial for some chemo-sensitive patients, the overall long-term efficacy remains inadequate [24, 25]. Therefore, alternative treatment approaches that incorporate the molecular or genetic characteristics of PMBCL are required. In particular, the frequently detected genetic alteration at the 9p24.1 locus is associated with programmed death-ligand 1 overexpression, indicating that ICIs may be effective against PMBCL. Indeed, based on the efficacy and safety of pembrolizumab in the phase 1 B KEYNOTE- 013 and phase 2 KEYNOTE- 170 trials, ICI has been approved by the FDA for the treatment of R/R PMBCL [26, 27]. However, real-world studies on ICI therapy in PMBCL are limited, and existing studies suggest that ICI therapy has modest efficacy. We evaluated the use of ICI in RR-PMBCL in the current study and found its efficacy to be suboptimal. Furthermore, biomarkers for predicting responses need to be identified (Table 5). Recent significant advancements and successes in cellular therapy have substantially expanded the treatment options for RR-PMBCL [28–30]. Nevertheless, there is currently no consensus on the optimal therapy for RR-PMBCL, highlighting the need for research on the sequence of salvage treatments and appropriate treatment selection based on patient characteristics and the nature of the disease.

**Table 5:** Summary of studies on use of immune checkpoint inhibitors to treat relapsed or refractory primary mediastinal large B-cell lymphoma

Study	Patients	Study type	Front-line regimen	Prior transplantation	Prior lines of treatment (median)	ICI	ORR	CR	Number of treatment (median)	Survival outcomes
KEYNOTE- 013	21	Phase 1b	R + CTx (100%)	42.9%	3 (range, 2–9)	Pembrolizumab	48%	33%	N/A	1 yr PFS 47% 1 yr OS 65%
KEYNOTE- 170	53	Phase 2	R + CTx (100%)	26.4%	3 (range, 2–8)	Pembrolizumab	41.5%	20.8%	N/A	Median PFS 4.3 mon Median OS 22.3 mon
CheckMate 436	30	Phase 1/2	R + CTx (100%)	13.3%	2 (range, 2–5)	Nivolumab	73.3%	40.0%	5 (range, 1–35)	2 yr PFS 55.5% 2 yr OS 75.5%
SJ Kim et al	4	Retrospective	N/A	50%	4 (range, 3–6)	Pembrolizumab	50%	0%	2.5 (range, 1–12)	N/A
Y Qin et al	4	Retrospective	N/A	N/A	N/A	Toripalimab Pembrolizumab Nivolumab Sintilimab	100%	100%	N/A	Median PFS: Patient 1: 21.2 mon Patient 2: 21.6 mon Patient 3: 22.9 mon Patient 4: 29.2 mon
B Casadei et al	27	Retrospective	MACOP-B ± R	N/A	3 (range, 2–8)	Pembrolizumab Nivolumab	N/A	37%	N/A	N/A
This study	12	Retrospective	R + CTx (100%)	8.3%	3 (range, 1–6)	Pembrolizumab Nivolumab	58.3%	25%	3 (range, 1–24)	1 yr PFS 20% 1 yr OS 54%

*Abbreviations: ICI* immune checkpoint inhibitor, *ORR* objective response rate, *CR* complete remission, *R* rituximab, *CTx* chemotherapy, *N/A* not available, *PFS* progression-free survival, *OS* overall survival, *MACOP-B* methotrexate, doxorubicin, cyclophosphamide, vincristine, prednisone, and bleomycin; mon, months

In conclusion, our study demonstrates the efficacy of front-line R-CHOP in patients with ND-PMBCL. Our findings support the proactive implementation of consolidative RT in patients with initially bulky disease or a DS of 4 on EOT PET-CT. The poor prognosis of patients with RR-PMBCL was confirmed in this study. Although ICI may be an option for salvage treatment, its efficacy remains suboptimal. Research efforts are increasingly focused on improving the survival outcomes for patients with RR-PMBCL, and where feasible, the proactive application of novel cellular therapies, such as chimeric antigen receptor T-cell therapy, is warranted.

## Data Availability

No datasets were generated or analysed during the current study.
